# Supplementary material for: Individual and familial factors predict formation and improvement of adolescents’ academic expectations: A longitudinal study in Sweden
Source: PLoS One. 2020 Feb 24;15(2):e0229505. doi: 10.1371/journal.pone.0229505 (PMC7039510; doi:10.1371/journal.pone.0229505)
Supplement: S1 Table — (DOCX) [file pone.0229505.s001.docx]

**Table S1** **Odds Ratio and 95% confidence intervals for lowering own academic expectations among adolescents reporting a definite academic expectation at baseline according to family and individual predictors (N=1,577)**

| **Variable** | **Model 1^a^** | **Model 2^b^** |
| --- | --- | --- |
|  | **OR (95% CI)** | **OR (95% CI)** |
| Family support for learning (continuous) | 0.89 (0.72-1.10) | 0.91 (0.72-1.15) |
| Parental expectations university vs. other | 0.34 (0.26-0.44) | 0.35 (0.26-0.48) |
| Future aspirations and goals (continuous) | 0.69 (0.56-0.85) | 0.73 (0.58-0.91) |
| Grades (continuous) | 0.95 (0.94-0.96) | 0.94 (0.93-0.96) |
| Identity synthesis (continuous) | 0.95 (0.80-1.12) | 0.96 (0.80-1.16) |
| CES-DC (ref=high score for depressive symptoms) | 0.70 (0.48-1.02) | 0.66 (0.44-0.99) |
| SDQ total (ref=high score for total difficulties) | 0.67 (0.44-1.01) | 0.65 (0.42-1.00) |

*OR=odds ratio, CI=confidence interval, CES-DC= Center for Epidemiological Studies Depression scale for Children, SDQ= Strengths and Difficulties Questionnaire.*

*^a^ Model 1 is unadjusted*

*^b^ Model 2 is adjusted* *for child’s gender, living arrangement, parental education, and parents’ country of birth.*
